# Prophylactic Cervical Cerclage in Cases Following Cervical Conization at a Japanese Perinatal Center

**DOI:** 10.7759/cureus.54639

**Published:** 2024-02-21

**Authors:** Yuria Haruna, Yoshie Shibata, Shunji Suzuki

**Affiliations:** 1 Obstetrics and Gynecology, Japanese Red Cross Katsushika Maternity Hospital, Tokyo, JPN

**Keywords:** japan, chorioamnionitis, cervical conization, preterm delivery, prophylactic cervical cerclage

## Abstract

The objective of this study was to re-examine the effect of cerclage on the possible factors associated with preterm delivery in women who had cervical conization. This was a retrospective cohort study comparing the obstetric outcomes of women with or without prophylactic cervical cerclage in pregnancy following a prior conization and managed at our institute between 2004 and 2023. In this study, there were 75% of pregnant women with a history of cervical conization. In 13 women of these (17%), prophylactic cervical cerclage was performed at 12-17 weeks' gestation. The incidence of preterm delivery was 15 (9/62) and 31% (4/13, p = 0.38) in cases with and without cervical cerclage, respectively. The prevalence of histological chorioamnionitis (CAM) in cases of preterm delivery following cervical cerclage was 100%. Prophylactic cervical cerclage in the cases following conization did not contribute to the prevention of preterm delivery associated with the development of CAM.

## Introduction

Cervical conization has been considered to be associated with an increased risk of cervical insufficiency [1] because there have been some observations of the relation between the history of cervical conization and the increased prevalence of preterm delivery and preterm premature rupture of the membranes (PPROM) [2,3]. Cervical cerclage is one of the common procedures performed for the treatment of cervical insufficiency; however, a recent review showed that cervical cerclage is associated with an increased risk of preterm delivery and PPROM in pregnancies that underwent conization [4-8]. A possible reason for the observation was suggested to be the presence of chorioamnionitis (CAM) [4,9], cervical lacerations, and suture displacement following cervical cerclage [9]. In addition, the incidence of these complications has been observed to vary widely with regard to the indications and timing for cerclage [10]. Therefore, the objective of this study was to re-examine the effect of cerclage and the possible factors associated with preterm delivery in women who had cervical conization.

## Materials and methods

The study protocol was approved by the Ethics Committee of the Japanese Red Cross Katsushika Maternity Hospital (K2018-27). In our institute, informed consent for analysis using a retrospective database was obtained from all pregnant women.

This was a retrospective cohort study comparing obstetric outcomes of women with or without cervical cerclage following prior recent conization of those managed at our institute between 2004 and 2023. During the period, 86 pregnant women with a history of cervical conization. The study was conducted on 75 women excluding 11 who had miscarriages at < 12 weeks' gestation. Based on informed consent, prophylactic cervical cerclage was performed in 13 women of these (17%, 11 cases of Mcdonald and 2 cases of Shirodkar cerclage) with no uterine contractions or progression of cervical shortening at 12-17 weeks gestation. We examined their medical chart as follows: age, parity, and cervical length at 12-16 weeks gestation, gestational age at delivery, the presence or absence of CAM based on histological examination, the presence or absence of cervical lacerations, and the presence or absence of suture displacement following cervical cerclage. Cervical lacerations or suture displacement were diagnosed by transvaginal ultrasound findings during prenatal visits or at the time of delivery, and one case of suture displacement was confirmed by pelvic examination.

In our institute, in cases of preterm delivery, microscopic histological analyses of the placenta were routinely performed for the diagnosis of the presence of CAM. In this study, the severity of CAM was determined according to Blanc’s criteria based on the degree of maternal polymorphonuclear lymphocyte infiltration into either the subchorionic space (stage I: intervillositis), the intervillous space (stage II: chorionitis), or the amniotic cavity (stage III) [11]. We assessed the women as having CAM in cases of stage II or III.

Data are presented as numbers (percentage, %). Statistical analyses were performed with the statistical software SAS version 8.02 (SAS Institute, Cary, NC, USA). Differences with p < 0.05 were considered significant.

## Results

Table 1 shows the clinical characteristics of pregnant women with or without cervical cerclage following a prior conization. There were no significant differences in these variables such as maternal age, parity, or preoperative cervical length between the two groups.

**Table 1:** Clinical characteristics of pregnant women with or without cervical cerclage following prior conization Data are presented as numbers (percentage, %). The differences were not statistically significant.

	Cerclage		P-value
	No	Yes	
Total number	62	13	
Maternal age			
≥ 35 years	39 (63)	8 (62)	0.93
Nulliparity	28 (45)	5 (38)	0.66
Cervical length at 12-16 weeks			
≥ 25 mm, < 35 mm	30 (48)	7 (54)	0.76
< 25 mm	6 (18)	1 (8)	0.91

Table 2 shows the presence of preterm delivery, histological CAM, cervical lacerations, and suture displacement in cases of cervical cerclage in pregnant women with or without cervical cerclage following a prior conization. The incidence of preterm delivery was 15 (9/62) and 31% (4/13, p = 0.38) in cases with and without cervical cerclage following a prior recent conization, respectively. There was no difference in the gestational age of delivery between the two groups in cases of preterm delivery. The prevalence of histological CAM in cases of preterm delivery following cervical cerclage was 100%; however, the difference in the prevalence of CAM did not reach statically significance compared to that in cases without cerclage (vs. 44%, p = 0.06). In one case, Mcdonald cerclage was diagnosed as suture displacement in labor and the case was delivered a few hours after the diagnosis.

**Table 2:** Preterm delivery, histological chorioamnionitis, cervical lacerations, and suture displacement in cases of cervical cerclage in pregnant women with or without cervical cerclage following prior conization Data are presented as numbers (percentage, %). The differences were not statistically significant.

	Cerclage		P-value
	No	Yes	
Total number	62	13	
Preterm delivery	9 (15)	4 (31)	0.38
Gestational age at delivery			
34 - 36 weeks	5 (8)	2 (15)	0.6
28 - 33 weeks	3 (5)	1 (8)	0.67
< 28 weeks	1 (2)	1 (8)	0.22
Histological chorioamnionitis in cases of preterm delivery	4 (/9, 44)	4 (/4, 100)	0.06
Cervical lacerations in cases of preterm delivery	0 (/9, 0)	0 (/4, 0)	1
Suture displacement in cases of preterm delivery	-	1 (/4, 25)	
Cesarean delivery at term	15 (29)	3 (9)	1

## Discussion

In this study, the incidence of preterm delivery was 15% and 31% in cases with and without cervical cerclage following prior recent conization, respectively. Although any significant differences were not obtained, probably due to the small study, it is suggested that prophylactic cervical cerclage in the cases following conization is associated with the development of CAM and does not contribute to the prevention of preterm delivery.

To date, the performance of cerclage in pregnant women with a history of conization has not been performed based on consistent evidence [4-8]. Our results also failed to find the effectiveness of prophylactic cervical cerclage in cases following conization. In an earlier study in Japan, there seemed to be no difference in the incidence of preterm delivery in women with or without prophylactic cerclage although they did not indicate the timing of cerclage [6]. In another earlier study, prophylactic cerclage has been recommended to be performed more sparingly in patients with a history of conization because cerclage tends to induce uterine contractions [12]. In addition, some studies have suggested that cervical cerclage will increase the risk of preterm delivery in patients with a cervical length of < 25 mm on ultrasound [13]. Whereas cerclage has been reported to be effective in cases with a short cervix without cervical inflammation, it has been observed to be deleterious in patients with inflammation [13,14]. These findings support that cerclage causes preterm delivery by inducing inflammation and CAM. Therefore, to examine the effect of prophylactic cervical cerclage in cases following conization, the subgroup analysis according to the different variables, such as the condition of the uterine cervix, may be required.

It has been pointed out that a history of conization itself may also induce inflammation of the cervix [15]. The cervical mucus plug can prevent ascending infection; however, conization has been suggested to cause a loss of the cervical mucus plug function [16,17]. Based on current results, at the very least, cerclage will not be able to prevent preterm delivery among patients who had been treated by conization.

We understand that there are some serious limitations in this study except the small retrospective study. As previously mentioned, the subgroup analysis according to the different variables may be required in this study. However, the small sample size prevented conducting subgroup analysis according to the different clinically important variables such as cervical condition and/or CAM. In addition, we could not analyze the risk of preterm delivery associated with the timing and/or the condition of the cerclage. Another major bias was that the decision to perform cerclage during the study period was made by informed consent based on the discretion of the individual physician. Therefore, to accurately assess the effect of the cerclage in the cases following conization on the prevention of preterm delivery, a large prospective study, in which cases are assigned to the same conditions, may be necessary.

## Conclusions

The performance of cerclage in pregnant women with a history of conization has not been performed based on consistent evidence. The current results will indicate that prophylactic cervical cerclage in the cases following conization is associated with the development of CAM and does not contribute to the prevention of preterm delivery.

## References

[1] Rincon M, Pereira LM (2012). Ambulatory management of preterm labor. Clin Obstet Gynecol.

[2] Albrechtsen S, Rasmussen S, Thoresen S, Irgens LM, Iversen OE (2008). Pregnancy outcome in women before and after cervical conisation: population based cohort study. BMJ.

[3] Bjørge T, Skare GB, Bjørge L, Tropé A, Lönnberg S (2016). Adverse pregnancy outcomes after treatment for cervical intraepithelial neoplasia. Obstet Gynecol.

[4] Wang T, Jiang R, Yao Y, Huang X (2021). Can prophylactic transvaginal cervical cerclage improve pregnancy outcome in patients receiving cervical conization? A meta-analysis. Ginekol Pol.

[5] Park HS, Kim HS, Lee SA, Yoon J, Kim EH (2021). Prophylactic cerclage to prevent preterm birth after conization: a cohort study using data from the National Health Insurance Service of Korea. Yonsei Med J.

[6] Miyakoshi K, Itakura A, Abe T (2021). Risk of preterm birth after the excisional surgery for cervical lesions: a propensity-score matching study in Japan. J Matern Fetal Neonatal Med.

[7] Cho GJ, Ouh YT, Kim LY (2018). Cerclage is associated with the increased risk of preterm birth in women who had cervical conization. BMC Pregnancy Childbirth.

[8] Rafaeli-Yehudai T, Kessous R, Aricha-Tamir B (2014). The effect of cervical cerclage on pregnancy outcomes in women following conization. J Matern Fetal Neonatal Med.

[9] Kassanos D, Salamalekis E, Vitoratos N, Panayotopoulos N, Loghis C, Creatsas C (2001). The value of transvaginal ultrasonography in diagnosis and management of cervical incompetence. Clin Exp Obstet Gynecol.

[10] Mitra A, Kindinger L, Kalliala I, Smith JR, Paraskevaidis E, Bennett PR, Kyrgiou M (2016). Obstetric complications after treatment of cervical intraepithelial neoplasia. Br J Hosp Med (Lond).

[11] Blanc WA (1959). Amniotic infection syndrome: pathogenesis morphology, and significance in circumnatal mortality. Clin Obstet Gynecol.

[12] Zeisler H, Joura EA, Bancher-Todesca D, Hanzal E, Gitsch G (1997). Prophylactic cerclage in pregnancy. Effect in women with a history of conization. J Reprod Med.

[13] Macnaughton MC, Chalmers IG, Dubowitz V (1993). Final report of the Medical Research Council/Royal College of Obstetricians and Gynaecologists multicentre randomised trial of cervical cerclage. Br J Obstet Gynaecol.

[14] Sakai M, Shiozaki A, Tabata M (2006). Evaluation of effectiveness of prophylactic cerclage of a short cervix according to interleukin-8 in cervical mucus. Am J Obstet Gynecol.

[15] Wada Y, Takahashi H, Ogoyama M (2023). Uterine cervical conisation and chorioamnionitis: a nationwide observational study. BJOG.

[16] Becher N, Hein M, Danielsen CC, Uldbjerg N (2010). Matrix metalloproteinases in the cervical mucus plug in relation to gestational age, plug compartment, and preterm labor. Reprod Biol Endocrinol.

[17] Hein M, Petersen AC, Helmig RB, Uldbjerg N, Reinholdt J (2005). Immunoglobulin levels and phagocytes in the cervical mucus plug at term of pregnancy. Acta Obstet Gynecol Scand.

